# Engineering Breathable Biodegradable Multilayers via Solution Blow Spinning for Sustainable Food Packaging

**DOI:** 10.3390/polym18121500

**Published:** 2026-06-16

**Authors:** Nasrin Moshfeghi Far, Ana Kramar, Javier González-Benito

**Affiliations:** 1 Department of Materials Science and Engineering and Chemical Engineering, Universidad Carlos III de Madrid, Av. Universidad 30, 28911 Leganés, Spain; 100458500@alumnos.uc3m.es; 2 Novel Materials and Nanotechnology Group, Institute of Agrochemistry and Food Technology (IATA), Spanish Council for Scientific Research (CSIC), Calle Catedrático Agustín Escardino Benlloch 7, 46980 Paterna, Spain; akramar@iata.csic.es

**Keywords:** solution blow spinning, cellulose acetate, poly(lactic acid), food packaging, multilayer materials

## Abstract

This study investigated porous materials based on cellulose acetate (CA), poly(lactic acid) (PLA), and their multilayer combinations fabricated by solution blow spinning (SBS) for potential food packaging applications. Single-layer neat polymers and multilayer structures (CA/PLA, CA/PLA/CA, and PLA/CA/PLA) were produced through sequential deposition, enabling control of layer arrangement while preserving high porosity. Attenuated total reflectance Fourier-transformed infrared spectroscopy, differential scanning calorimetry, and thermogravimetric analysis showed negligible polymer interdiffusion or specific intermolecular interactions, indicating that layer integration occurs mainly through physical contact and void filling rather than molecular mixing. Scanning electron microscopy analysis revealed that cellulose acetate possesses a highly porous, interconnected structure, whereas poly(lactic acid) exhibits a predominantly fibrous morphology with clearly distinguishable layers in multilayer systems. Mechanical testing demonstrated that poly(lactic acid) mats had higher stiffness and tensile strength, while cellulose acetate films were more flexible and compliant. Multilayer systems showed complex tensile behavior characterized by interfacial failure and limited load transfer, indicating no synergistic mechanical reinforcement between layers. Water vapor permeability remained high and narrowly distributed for all configurations (890–920 g·m^−2^·day^−1^), independent of layer sequence, reflecting the porous morphology. These values exceed those of conventional polymer packaging films, highlighting the suitability of the materials for breathable packaging. Overall, solution blow spinning enables scalable fabrication of biodegradable multilayer materials with tunable mechanical performance for sustainable food packaging applications requiring controlled moisture exchange.

## 1. Introduction

Several approaches can be followed when designing materials for food packaging. They typically involve the synthesis of new polymers, the modification of existing polymers by incorporating chemical additives or nanoparticles, and the development of polymer blends. In these cases, the material systems present unified properties that arise from the particular combination of properties provided by different functional groups or components. However, achieving the desired synergistic effects may require maintaining distinct functionalities within physically coupled materials, rather than blending properties into a single homogeneous system. Multilayer architectures represent one effective strategy to accomplish this.

Multilayer plastic films are prepared to combine the properties of different polymers in a single material, aiming to improve the final performance [1,2]. This technology is especially used in flexible packaging for food [3], pharmaceuticals [4], and cosmetics [5]. The idea is to integrate two or more layers of different polymer-based materials, each with a specific function for the required final application. Examples include oxygen, humidity, light barrier, mechanical strength, thermal sealability, transparency, or aesthetics, as well as compatibility with food or pharmaceutical products [2,3,4].

Nowadays, great efforts are being made to replace non-biodegradable single-use plastics for food packaging with biobased and biodegradable polymers. However, as in the case of common non-environmentally friendly synthetic plastics, a single material may not contain all the required properties for covering most of the functions addressed to extend the good condition of food for later consumption. Therefore, a combination of several biodegradable materials in the form of films coupled to form a multilayer system is a very attractive solution.

Among the most well-known biodegradable polymers whose combination of properties could represent a significant advancement in the performance of materials used for food packaging, cellulose acetate (CA) and poly(lactic acid) (PLA) gather great interest. Cellulose acetate is a biodegradable and semi-synthetic polymer derived from natural cellulose, typically sourced from wood pulp or cotton [6]. It is produced by acetylating cellulose with acetic anhydride and acetic acid in the presence of a catalyst, having a high ability for film and fiber formation [7]. Among its key features, the following stand out. It is biodegradable under enzymatic and hydrolytic conditions (the presence of water and an adequate pH); CA loses acetyl groups by hydrolysis to yield cellulose and acetic acid. After that, the regenerated cellulose can be degraded by microbial cellulase to yield oligosaccharides. CA presents optical clarity and a glossy finish, which is attractive for food presentation. In addition, it has good tensile strength and flexibility. It is resistant to oils and greases, which is beneficial for packaging fatty foods, and it is easily printable, allowing for high-quality labeling and branding directly on a film. However, it provides a moderate barrier to moisture and gases, and it is only stable at moderate temperatures, which makes it non-ideal for high-heat applications. Moreover, although CA has good tensile strength (30–74 MPa) and Young’s modulus (1.6–4 GPa), its flexibility can be conditioned by the final processed product [6]. In fact, cracking or tearing during packaging and handling could occur. On the other hand, due to its hydrophilic nature, CA can absorb moisture, leading to swelling or loss of mechanical integrity in humid environments. Therefore, its combination with other polymers that provide characteristics that cover those deficiencies would be very welcome.

In the last 20 years, PLA has received special attention for its potential use in food packaging [8]. PLA is a biobased, compostable thermoplastic derived mainly from corn starch and sugarcane. After fermentation of the bioproducts, lactic acid is produced, which is then used to yield PLA through direct polycondensation or ring-opening polymerization of lactide. PLA offers good optical clarity and mechanical behavior (tensile strength of 50–70 MPa, Young’s modulus of 3–4 GPa) [9], and printability, making it attractive for food packaging. However, its small amount of elongation may limit its use in applications that require high flexibility. In addition, PLA was accepted by the FDA (U.S. Food and Drug Administration) for use in food contact applications around 2002. PLA has moderate barrier properties to oxygen and carbon dioxide, but is relatively permeable to water vapor, which limits its use for high-moisture products unless combined with other materials. It performs well in thermoforming and extrusion but has low thermal resistance (T_g_ = 60 °C), which restricts its use in hot fill applications.

Considering the properties of CA and PLA concerning their potential use for food packaging, it seems that their combination in the form of a multilayer material could offer several advantages if optimizing functional properties and sustainability are looked for. In terms of barrier properties, CA offers good behavior concerning O_2_ and aromas [10,11] while PLA provides an acceptable barrier behavior against CO_2_ and water vapor, but is poor against O_2_ [12]. Therefore, the combination of these two polymers with overlapping layers can compensate for their weaknesses. Concerning the mechanical behavior, the PLA has good rigidity, but it is brittle. When using it in combination with CA, an improvement of global toughness is expected. Moreover, a multilayer design would allow adjusting the thickness of each layer to tailor the material performance as a function of its final application. Finally, considering the different physico-chemical nature of CA, and therefore different affinities for additives, more activities could be reached by the specific modification of each polymer. However, this aspect might be a serious concern since CA and PLA are not compatible at a molecular scale [13], so it might be necessary to find a way to improve the adhesion between layers. Beyond food packaging applications, CA/PLA systems have also been explored in other fields, such as biomedicine (scaffolds for tissue engineering), filtration membranes, and controlled drug delivery systems, where their biocompatibility and biodegradability are advantageous. However, across these applications, a recurrent limitation is the weak interfacial interaction between the two polymers, which often leads to phase separation, reduced mechanical integrity, or limited load transfer in composite or multilayer configurations. Various strategies have therefore been proposed in the literature to improve CA/PLA compatibility, including blending with compatibilizers, surface treatments, and chemical functionalization, although these approaches often add processing complexity or compromise biodegradability.

Currently, the most common techniques to produce multilayer plastic materials are coextrusion, lamination, and solvent casting, since they present high throughput and scalability [14]. However, in general, they do not allow creating porous or breathable functional layers that would be of high interest for fresh produce packaging (fruits, vegetables, and baked goods). Due to this, techniques that produce highly porous materials, even with control of morphology, are receiving special attention because, when the final thermoplastic material is required with reduced or no porosity, those techniques could be combined with other processes such as hot pressing. Among those techniques, electrospinning (ES) and solution blow spinning (SBS) are receiving special attention since materials constituted by layers of fine fibers are obtained and the deposition of layers can be carried out very easily through a consecutive steps process or additive manufacturing process. Although ES is a better-known process with which good control of morphology can be obtained, the necessity of applying a high electric field can reduce the range of polymer systems to be processed; moreover, in general, only low production rates are achieved [15]. On the other hand, SBS seems to be a convenient alternative when high production rates are preferred, although that means losing some morphological control. In SBS, instead of an electric field, a pressurized gas is used as the driving force to stretch a polymer solution to generate fibers while the solvent evaporates [16]. Using both techniques, ES and SBS, CA and PLA materials were prepared. For example, ES was used to prepare fibrous CA-based materials. It was demonstrated how the solvent system, solution concentration, and applied electrostatic field strength influence the morphological appearance and/or size of as-spun cellulose acetate (CA) products [17] suitable for applications in filtration and biomedical fields [18]. In the same way, nanofibrous PLA was prepared by ES for multiple purposes, including biomedical applications [19] and food packaging [20], among others. In the same way, SBS was recently used to prepare CA-based fibrous materials; however, spinnability is quite difficult if pure CA is required, reducing the much sought-after obtention of fiber films [21,22]. Due to this, other polymers were tested to co-spin CA to finally get materials mainly constituted by fibers [23]. In the case of PLA, spinnability through SBS is much easier, as more information can be found in the bibliography about PLA nanofibers prepared by SBS for multiple applications [24].

Materials prepared by ES or SBS, constituted by sequentially superimposed layers of CA and PLA, are scarcely reported in the literature. A.K.M. Mashud Alam et al. prepared multilayer sandwich-structured nanofiber membranes based on regenerated cellulose and poly(lactic acid) by electrospinning, demonstrating the possibility of tuning surface wettability through multilayer design [25]. However, despite these advances, analogous multilayer systems produced by SBS have not yet been investigated. The novelty of the present work lies not only in the fabrication of CA/PLA multilayer assemblies but also in the use of SBS as the processing route. Among the advantages mentioned above, the morphology, porosity, interlayer adhesion, and overall performance of multilayer structures can differ significantly depending on the fabrication technique employed. Therefore, results obtained for electrospun CA/PLA multilayers cannot be directly extrapolated to SBS-derived systems. Accordingly, the present work aims to prepare, for the first time, flexible multilayer systems based on sequentially stacked films of cellulose acetate and poly (lactic acid) films produced by solution blow spinning and to characterize their structural, thermal, mechanical, and barrier properties in the context of food-packaging applications. By investigating SBS-derived multilayer CA/PLA films, this study provides new insights into the relationship between processing route and material performance, while opening a pathway toward scalable, biodegradable, and breathable packaging materials.

## 2. Materials and Methods

### 2.1. Materials

Cellulose acetate (CA) with an average molecular weight (Mn ~30,000) and an acetyl content of 39.8 wt.% (St. Louis, MO, USA) was used. The degree of substitution was determined to be 2.45 according to the ASTM D871-96 standard [26]. The material was stored in sealed containers under laboratory conditions before use, and all solutions were prepared using the same batch to ensure consistency among samples. Poly(lactic acid) (Ref. code: PLA Polymer 7032D) was sourced from Nature Works LLC (Blair, NE, USA) through Resinex Spain, SL (Tarragona, Spain). The PLA had a glass transition temperature (T_g_) of 55–60 °C, a melting temperature (T_m_) of 160 °C, and a processing temperature range of 200–220 °C. Dichloromethane (99.8% purity, Sigma-Aldrich, St. Louis, MO, USA), isopropyl alcohol (propan-2-ol, 99.9% purity, Carlo Erba, St. Filadors, Barcelona, Spain), and acetic acid (99.5% purity, Panreac, Barcelona, Spain) were used as solvents for preparing the polymer solutions.

### 2.2. Sample Preparation

Both polymer solutions, CA and PLA, respectively, with concentrations of 10% (wt/v), were prepared. Cellulose acetate was dissolved in acetic acid (HAc), while poly(lactic acid) was dissolved in a binary solvent mixture of dichloromethane (DCM) and isopropanol (iPOH) at a volume ratio of 1:5 (*v*/*v*). Before magnetic stirring, the polymer–solvent mixtures were left for at least 2 h at room temperature to allow initial polymer swelling, which facilitated subsequent dissolution. Afterwards, the solutions were magnetically stirred overnight at room temperature until complete dissolution was achieved. All solutions were prepared under identical conditions to ensure reproducibility.

**Figure 1:**
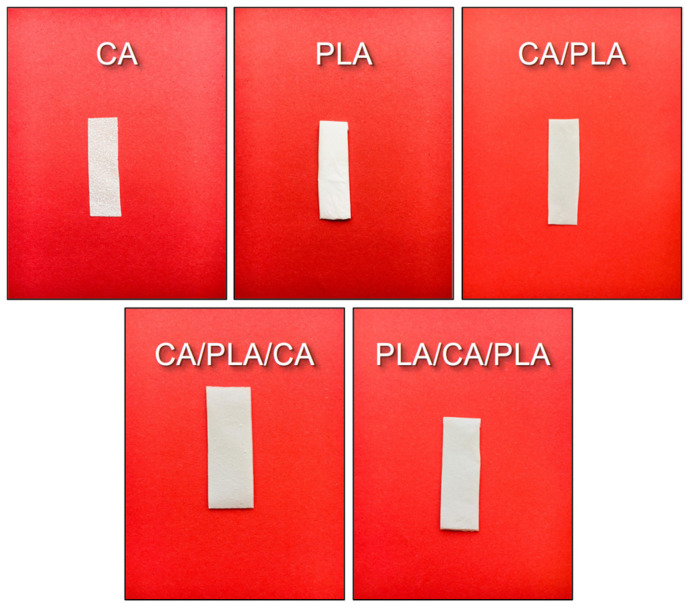
Representative photos of the differently prepared samples.

Polymer film samples were prepared via solution blow spinning (SBS) and collected on a rotating cylindrical drum covered with anti-adherent paper at a rotation speed of 250 rpm. The polymer solution in a syringe is injected into a capillary (0.6 mm for the inner diameter and 0.9 mm for the outer diameter) that constitutes the inner channel of a nozzle, which extends 2 mm beyond the nozzle, while pressurized air is passed through the outer channel of the nozzle (1.2 mm in diameter) with a flow of 15 mL/min. When the polymer solution reaches the exit of the nozzle, the pressurized air elongates it, forming thin fibers while the solvent evaporates. Finally, the fibers are deposited on the collector after 20 mL of solution is dispensed. A polymeric film of homogeneous thickness is obtained on the collector thanks to the motion of the nozzle at a constant rate perpendicular to the direction of rotation of the collector. To prepare the multilayer systems, polymer films were deposited on the collector one after another, consecutively, after changing the corresponding polymer solution in the syringe. Each layer was formed by processing 20 mL of solution. Table 1 includes sample codes, polymer components used, and the schemes showing the configuration of the prepared samples. Here, it should be highlighted that in multilayer samples, deviations from the expected additive thickness were observed (Table 1). This is attributed to partial compaction of previously deposited porous layers during subsequent SBS deposition, caused by the impingement of the gas jet and the deposition of the upper layer, which leads to partial collapse and densification of the highly porous fibrous structure. On the other hand, Figure 1 shows representative photos of the specimens taken from the different samples.

### 2.3. Methods and Equipment

To study the chemical structure, Attenuated Total Reflectance Fourier-Transform Infrared Spectroscopy (ATR-FTIR) was employed. The analysis was conducted using a Nicolet iS5 spectrometer (Thermo Scientific, Thermo Fisher, Madison, WI, USA) equipped with an attenuated total reflectance (ATR) device featuring a diamond window (GladiATR, PIKE Technologies, Madison, WI, USA). A total of 32 scans were performed using the Golden Gate ATR accessory with a diamond window to generate Fourier-transformed infrared spectra. The spectra were recorded across a wavelength range of 400–4000 cm^−1^ with a resolution of 4 cm^−1^.

The morphology of the prepared materials was inspected through the observation of cross-sections generated from the freeze fracture of specimens. A TENEO-FEI field emission scanning electron microscope (FESEM, FEI Company, Hillsboro, OR, USA) at an acceleration voltage of 10 kV was used. Samples were vertically mounted on SEM stubs specially manufactured for that purpose. To avoid electrostatic charge accumulation on the surface of the samples, a gold coating was carried out using a Leica EM ACE200 low-vacuum coater (Leica Microsystems, Wetzlar, Germany), current of 30 mA for 40 s. Images were obtained from the use of the signal coming from both secondary and backscattered electrons. Analysis of images was done using the open-access software ImageJ 1.51j8 (National Institutes of Health, Bethesda, MD, USA).

The thermal behavior of multilayer polymer films was studied by differential scanning calorimetry (DSC) and thermogravimetric analysis (TGA). In the case of DSC, a Mettler Toledo 822e calorimeter (Mettler-Toledo GmbH, Greifensee, Switzerland) was used under a nitrogen atmosphere. Although samples were subjected to two thermal cycles of heating and cooling, only the first heating was used to compare the thermal behaviors of the different materials, since only information from the as-prepared materials is required. This first heating cycle consisted of a temperature ramp from 20 °C to 210 °C at 10 °C/min. Thermal transitions, including the glass transition, melting, and crystallization temperatures, were evaluated during the heating cycle. The degree of crystallinity (*χ*) was calculated from the heating scans using the equation:

(1) $$\chi =\frac{∆H_{real}\cdot (1-x)}{∆H_{100}}$$

(2) $$∆H_{real}={∆H}_{m}-{∆H}_{cc}$$

where x is the weight fraction of the polymer for which the degree of crystallinity is not being calculated, Δ*H_m_* is the melting enthalpy, Δ*H_cc_* is the cold crystallization enthalpy, and Δ*H*_100_ represents the melting enthalpy of a 100% crystalline polymer. To identify the polymer for which *χ* will be obtained, a subscript will be added, PLA or CA, to identify a particular polymer. For these calculations, the reported values of 93.6 J/g and 58.8 J/g were used for 100% crystalline PLA [9] and cellulose triacetate [27], respectively.

On the other hand, to carry out TGA, a PerkinElmer STA 6000 thermogravimetric analyzer (PerkinElmer Inc., Waltham, MA, USA) was used. Approximately 20 mg of each sample was placed in a high-temperature-resistant crucible and subjected to controlled heating. The temperature program was set from 30 °C to 700 °C at a constant heating rate of 10 °C/min, under a nitrogen atmosphere with 20 mL/min flow to prevent oxidative degradation.

The mechanical behavior of the materials was evaluated using a Universal Testing Machine, Microtest DT/005/FR (Microtest S.A., Madrid, Spain), equipped with a 50 N load cell. For each sample, the average length, width, and thickness were determined. Thickness measurements were obtained by averaging three readings taken at different points along the specimen using a Digimatic micrometer (Mitutoyo Corporation, Barcelona, Spain), with an accuracy of ±1 μm, while length and width were measured using a caliper. Each specimen measured 35–45 mm in length and 7–9 mm in width, while when they were positioned in the universal testing machine, the grip-to-grip distance was 20–21 mm, also measured using a caliper. Specimens were tested in a uniaxial tensile configuration at a loading rate of 5 mm/min. Mechanical tests were conducted six times for each sample, using specimens cut so that the major axis corresponds to the direction of collector rotation during the SBS process. Before positioning the specimens in the testing machine, rubber pieces were attached to the surface of the clamps to prevent slippage during the tensile tests.

Porosity was determined following a gravimetric method previously reported by our group [28] through Equation (1), which is based on comparing the apparent (sample) density of the fibrous material, *δ_s_*, to the true (solid) density of the polymer, δ, from which the fibers are made:

(3) $$Porosity\left(\%\right)=\left(1-\frac{\delta_{s}}{\delta }\right)\times 100$$

First, the mass of fibrous specimens is measured in an analytical balance. Then, the volume of the specimens is obtained, knowing the dimensions (length, width, and thickness) measured with a caliper (±0.01 mm accuracy) and a micrometer (Digimatic, Mitutoyo Corporation, with an accuracy of ±1 μm). Finally, the apparent density is calculated as an average obtained from the densities calculated through the quotient between the mass of each specimen and its corresponding volume. In the case of the true density, a simple rule of mixtures was used (Equation (4)):

(4) $$\delta =\phi_{CA}\cdot \delta_{CA}+\phi_{PLA}\cdot \delta_{PLA}$$

where *ϕ_CA_* and *ϕ_PLA_* are the volume fractions of cellulose acetate and poly(lactic acid) in the samples, respectively, and δ_CA_ = 1.3 g·cm^−3^ [29] and δ_PLA_ = 1.24 g·cm^−3^ (information given by the supplier) are their densities.

**Figure 2:**
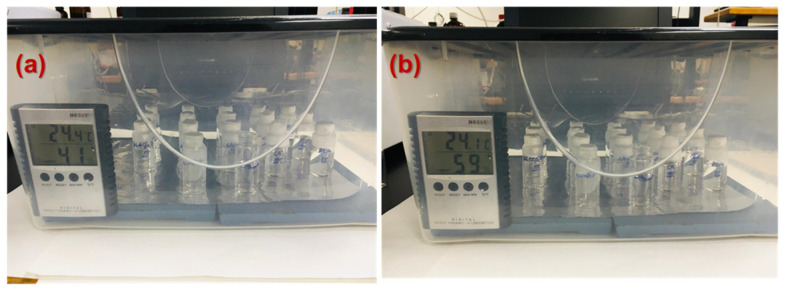
Representation of the water permeability test. (**a**) Before and (**b**) after exposure to controlled humidity conditions.

Water vapor permeability was studied through the determination of the water vapor transmission rate (WVTR), inspired by the method provided in the standard ISO 2528:2017 [30], which was slightly modified considering the kind of specimens prepared in this work. Seventeen glass vials were filled with distilled water. After that, one of them was sealed with Parafilm, another was left uncovered to use it as a control, and the rest of the vials were covered with the materials prepared in this work. The initial weight (*w_i_*) of each vial was recorded before placing them in a controlled atmosphere chamber at ~24 °C and 41% relative humidity for 24 h (Figure 2a). After the exposure period selected, the vials were reweighed (*w_f_*) under conditions of ~24 °C and 59% relative humidity to assess water loss (Figure 2b). Finally, the water vapor permeability or water vapor transmission rate (WVTR) is obtained as the weight loss (*w_i_* − *w_f_*) per surface unit or relative to the surface area (A) through which water vapor transmission occurs (Equation (5)). All samples were tested in triplicate, giving the final value of WVTR in g·m^−2^·day^−1^ as the corresponding mean value:

(5) $$WVTR=\frac{w_{i}-w_{f}}{A}$$

To determine whether there are statistically significant differences between the means of five independent groups of data corresponding to eight samples studied, one-way ANOVA analysis of variance was performed with a significance level of 0.05.

## 3. Results and Discussion

### 3.1. Chemical Structure of Multilayer Materials

**Figure 3:**
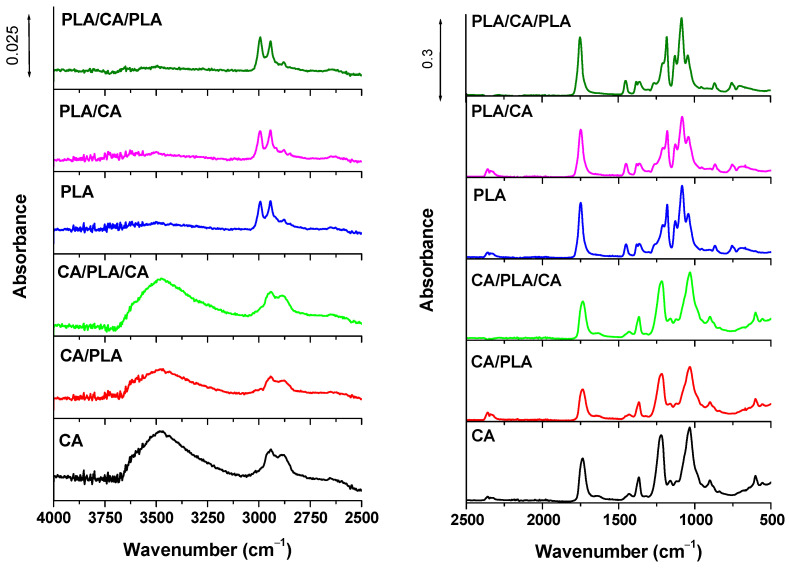
ATR-FTIR spectra of the samples under study.

The chemical structure was analyzed by ATR-FTIR (Figure 3). As expected, the IR bands observed correspond to those associated with the polymer layer directly in contact with the diamond window of the ATR device. The reason is that when using ATR, the FTIR radiation does not penetrate the sample much to reach further layers from the excitation window. The IR penetration in a polymer is usually within the range 0.5–5 µm [31], and the polymer layers of the systems studied are thicker than 20 µm. When the layer directly in contact with the diamond window is CA, PLA bands are absent, while the bands reflecting the cellulose backbone and the acetyl functional groups are observed, highlighting the bands at 1740 cm^−1^ and 1222 cm^−1^ of the carbonyl stretching from the acetyl groups (C=O) and the ester stretching (C-O), respectively [32]. The broad band centering at around 3400 cm^−1^ indicates the remaining -OH groups corresponding to the absence of total acetylation and some water absorbed. On the other hand, when the layer directly in contact with the diamond window is PLA, CA bands are absent, while typical PLA infrared absorption bands [33] corresponding to the carbonyl group at 1750 cm^−1^, the ester group at 1180 cm^−1^ and 1084 cm^−1^, and the carbon-carbon stretching of the C-CH_3_ group at 1043 cm^−1^ are observed without showing any representative variations in terms of their ratios. All these results point out that there is no full interpenetration of fibers of the different polymers, nor specific interactions with each other.

### 3.2. Morphology and Cross-Section Analysis of Multilayer Materials

**Figure 4:**
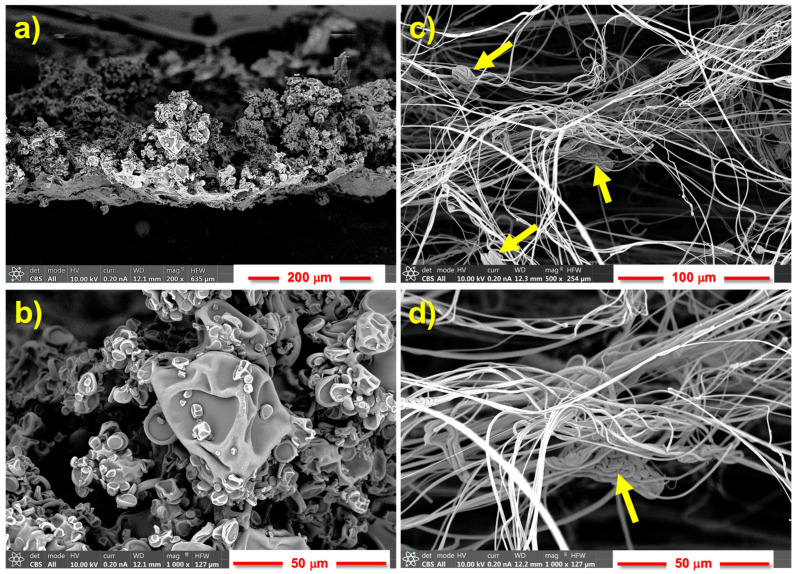
Representative cross-section scanning electron microscope images of the neat CA sample (**a**,**b**) and the neat PLA sample (**c**,**d**). Arrows point out occasional bead-like features or thickened regions.

The cross-sections of all samples were inspected using scanning electron microscopy. In Figure 4a,b, the morphology corresponding to the sample CA is shown. It can be observed that the material is primarily composed of irregular droplets with a relatively broad size distribution that is commonly a consequence of the well-known poor spinnability of CA [34,35]. Among the reasons to explain this morphology, it can be highlighted that cellulose acetate has a stiff backbone with limited chain flexibility required for fiber formation. In addition, strong intermolecular interactions can appear since even though the hydroxyl groups are partly substituted with acetyl groups, CA still forms strong hydrogen bonds, as reflected in the position of the carbonyl IR absorption band at wavenumbers below 1740 cm^−1^ (Figure 3). These interactions hinder chain sliding, reducing the ability of the polymer to elongate under shear and stress during spinning. Another possibility is the use of a solution with a high surface tension relative to its viscosity [22], causing the jet to break or contract into droplets, making it difficult to maintain continuous defect-free fibers [18,36,37]. In fact, acetic acid has a relatively high surface tension (~28 mN/m) and low viscosity (~1.20 mPa·s) at room temperature in comparison with other solvents commonly used for spinning processing [38].

In Figure 4c,d, the morphology corresponding to the sample PLA is shown. The image shows a non-woven mat composed of randomly oriented fibers, as was obtained previously for the same system using similar processing conditions [33,39]. The fibers appear to vary in diameter, ranging from a submicron to a few microns (mean diameter approximately 700 nm), where some thinner fibers intertwine with thicker ones, resulting in a heterogeneous texture. This result is usually attributed to variations in local airflow strength [40,41]. The fibers mostly appear smooth, without obvious granularity or rough surface features, indicating good polymer chain packing upon solidification. Interconnected pores are evident between the fibers. The porosity was estimated at ~90% with a pore size distribution that is irregular, consistent with the chaotic deposition from a turbulent air jet. Furthermore, occasional bead-like features or thickened regions are visible along certain fibers (see inserted arrows in Figure 4). These could arise from jet instabilities or incomplete solvent evaporation. The fibers are randomly oriented rather than aligned, forming an isotropic mat, expected in blow spinning, where the deposition is governed by turbulent airflow. Since PLA is dissolved in a volatile solvent, during flight, rapid solvent evaporation causes the polymer solution to solidify into the fibers. If evaporation is incomplete before deposition, residual solvent can cause fiber fusion or bead formation, consistent with the visible thick spots.

**Figure 5:**
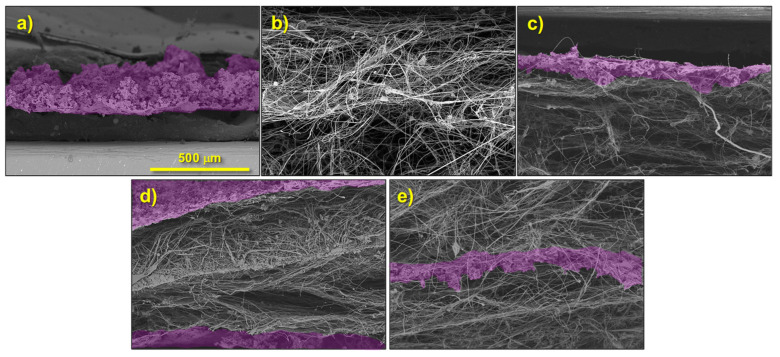
Cross-sectional SEM micrograph of (**a**) cellulose acetate (CA); (**b**) PLA; (**c**) CA/PLA bilayer; (**d**) CA/PLA/CA trilayer; and (**e**) PLA/CA/PLA trilayer films fabricated by solution blow spinning (SBS). CA layers are colored purple to better distinguish them from PLA layers.

In Figure 5, images of the cross-section of the five systems under study are considered to be compared with each other. The primary difference between the morphologies of the monolayer systems is thought to be governed by the viscosity of the polymer solution, the volatility of the solvent, and the tendency for phase separation. Specifically, the CA solution exhibits a viscosity above 0.4 Pa·s [22], whereas the PLA solution has a viscosity of approximately 0.1 Pa·s under comparable conditions. Despite its substantially higher viscosity, the CA solution did not produce fibrous structures, indicating that viscosity is not the sole parameter governing morphology formation in these systems. This observation supports what was mentioned above, that other factors, such as solvent evaporation rate, polymer–solvent interactions, chain entanglement characteristics, and phase-separation phenomena, play a significant role in determining the final morphology. Therefore, while viscosity is undoubtedly an important parameter, the present results highlight the need to consider the combined influence of multiple solution and processing variables.

In the case of CA film (Figure 5a), a highly porous, foam-like morphology is observed where a continuous, dense polymer matrix permeated by numerous irregular, interconnected pores arises mainly from beads completely coalesced. On the other hand, as explained above, a fibrous morphology was obtained for the PLA monolayer (Figure 5b), where a network of randomly oriented, solid submicron fibers (~700 nm) with inter-fiber pores is observed. Figure 5c shows two distinct layers: a bottom layer of porous CA film (foam-like) and a top layer of fibrous PLA mat. In the three-layer porous CA film (bottom)/fibrous PLA mat/porous CA film (top) (Figure 5d), the PLA layer, being relatively dry and fibrous, is clearly distinguished and fully sandwiched between two CA layers, acting as a structural separator. The CA-PLA interfaces show the characteristic CA foam structure transitioning sharply into the PLA fibrous network, where the high surface area of the PLA mat may allow the second CA jet to partially penetrate or fill the void spaces of the top of the PLA mat. Finally, in the three-layer fibrous PLA mat (bottom)/porous CA film/fibrous PLA mat (top) (Figure 5e), the CA layer is fully sandwiched between two PLA layers, with a very high structural contrast, a porous, dense foam material (CA) confined between two highly porous and open non-woven fiber networks (PLA). For a multilayer system with two outer PLA layers and an inner cellulose acetate (CA) layer, the fibrous PLA layers serve as the main structural support, while the CA foam core is largely enclosed within them. Here, it is important to emphasize that the absence of a pause was intentional, aimed at achieving functional advantages. This approach enhanced interlayer adhesion and minimized the risk of delamination by enabling controlled interfacial interpenetration without compromising the identity of each layer. Moreover, this was effectively controlled through processing parameters rather than relying on time delays, as evidenced by the well-preserved morphologies and defect-free interfaces observed in the SEM images.

In the multilayer systems, two distinct layers are observed: porous CA films (foam-like) and layers of fibrous PLA mat. It seems there is a minimal inter-layer mixing caused by the high difference between the solvents used in each case (HAc for CA and DCM-iPOH for PLA). When a PLA layer is spun immediately after the CA layer, which is considered non-wet or slightly wet, since the pressurized air usually allows removing all or almost all solvent during the SBS process (no traces of HAc were detected in the FTIR spectra, Figure 3), the PLA solvent (mixture iPOH/DCM) can cause minor surface swelling/dissolution of the CA, leading to very little interfacial entanglement at the interface. In addition, the high production rate of SBS often ensures that the first layer is sufficiently solidified (though possibly still containing residual solvent) before the next solution is applied [40,42], preventing excessive inter-layer diffusion and maintaining sharp interfaces. However, when the CA layer is spun immediately after the PLA layer, interpenetration is more feasible, yielding better compatibility that should, for instance, be translated into a better transmission of mechanical loads.

### 3.3. Thermal Properties of Multilayer Materials

**Figure 6:**
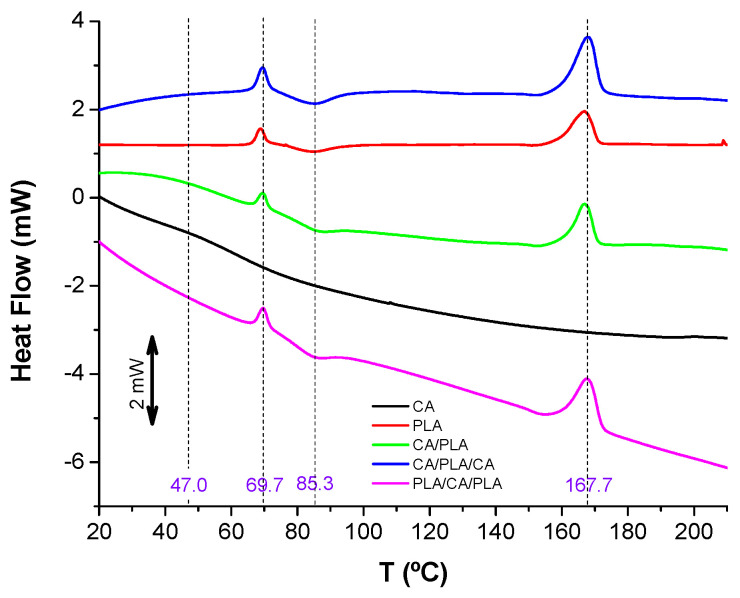
DSC traces for the first heating of the 5 SBS materials under study.

The thermal behavior of the SBS systems was studied by DSC. Figure 6 shows the thermograms corresponding to the first heating (without erasing thermal or processing history) of the five systems under study. After carefully analyzing the DSC traces, it can be concluded that thermal transitions of pure components, within the range of temperatures considered, are not altered by the presence of the other in the multilayer systems (Table 2), which confirms that there is no significant polymer chain interdiffusion (solubility) between polymers. The only possible integration between layers must be due to void filling.

The broad and smooth endodermic transition for CA observed from ~47 °C (Figure 6) can be caused by the evaporation or desorption of water present in the sample, as it is observed in the FTIR spectrum (Figure 3). On the other hand, the effect of the multilayer structure on PLA crystallinity does not follow a clear general trend. While the CA/PLA and CA/PLA/CA systems exhibit lower crystallinity values than neat PLA (Table 2), the PLA/CA/PLA configuration shows a slight increase in crystallinity. These results suggest that the crystallization behavior of PLA is influenced not only by the presence of cellulose acetate but also by the specific layer arrangement and interfacial architecture of the multilayer system. The different configurations may affect chain mobility and crystal growth during processing, leading to variations in the final degree of crystallinity [43]. Therefore, the observed changes cannot be attributed solely to interfacial heterogeneous nucleation, and additional studies would be required to clarify the mechanisms governing crystallization in these multilayer structures.

### 3.4. Thermodegradation Studies

**Figure 7:**
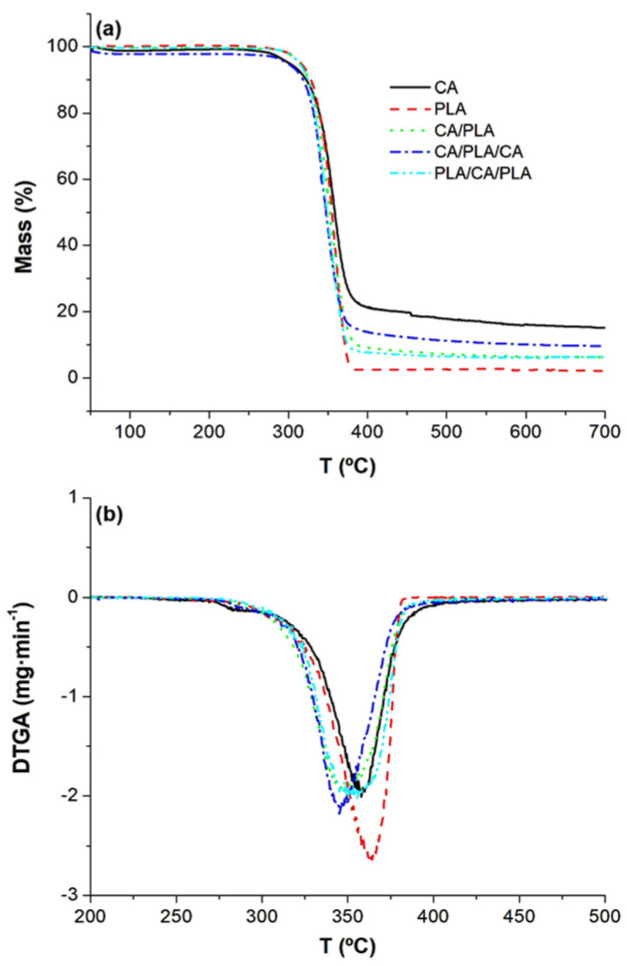
(**a**) Thermogravimetric analysis (TGA), and (**b**) derivative thermogravimetric analysis (DTGA) curves for pure SBS cellulose acetate (CA), pure SBS poly(lactic acid) (PLA), and the multilayer systems prepared by SBS.

Figure 7 presents the thermogravimetric analysis (TGA, Figure 7a) and derivative thermogravimetric analysis (DTGA, Figure 7b) curves for pure SBS cellulose acetate (CA), pure SBS poly(lactic acid) (PLA), and the multilayer systems prepared by SBS. All samples exhibit a single dominant degradation step occurring between approximately 300 and 400 °C, indicating that thermal decomposition proceeds mainly through one primary mechanism without secondary degradation stages. In the case of neat PLA, the negligible mass loss below 250 °C points to the absence of residual solvent or physically absorbed moisture. In contrast, the remaining samples show a small mass loss, which can be attributed to the presence of CA and the associated low water content, as also suggested by FTIR and DSC analyses.

Neat PLA shows an abrupt mass loss starting at approximately 330 °C and reaching its maximum degradation rate near 360–370 °C (Figure 7b). The degradation process is sharp and nearly complete, leaving less than 5 wt% residue at 700 °C. This behavior is consistent with the well-established thermal degradation mechanism of PLA, which involves random chain scission, intramolecular transesterification, and depolymerization into lactide and other volatile oligomers [44,45]. The low char yield reflects the limited formation of stable carbonaceous structures during pyrolysis.

In contrast, neat CA exhibits a slightly broader degradation profile with a T_max_ located around 340–355 °C. The degradation onset appears marginally lower than that of PLA, which can be attributed to the deacetylation process preceding backbone scission. CA degradation typically proceeds via (i) elimination of acetic acid, (ii) cleavage of the glycosidic backbone, and (iii) progressive formation of carbonaceous residue [46,47]. Consequently, CA shows a significantly higher residual mass at 700 °C (≈15 wt%), indicating enhanced char formation capability compared to PLA.

The multilayer systems display intermediate thermal behavior between their constituent polymers. The onset degradation temperatures and T_max_ values are positioned between those of neat CA and PLA, suggesting that the overall thermal response is governed by the relative contribution of each component. Importantly, no additional degradation steps are observed, indicating the absence of new chemically distinct phases or strong covalent interactions formed during multilayer processing.

The DTGA curves (Figure 7b) provide further insight into degradation kinetics. PLA exhibits the most intense and narrowest peak, indicating rapid mass loss over a short temperature interval. In contrast, CA shows a broader and less intense peak, reflecting a more gradual degradation process. For the multilayer systems, the maximum degradation rate is reduced compared to neat PLA, and the DTGA peaks become slightly broader. This reduction in peak intensity suggests moderated degradation kinetics, likely associated with structural and interfacial effects. The absence of multiple DTGA peaks confirms that the degradation of the multilayer structures proceeds without phase-separated thermal events, supporting the conclusion that the layers remain physically distinct but thermally compatible.

Although the T_max_ values of the multilayer systems do not significantly exceed those of neat PLA, a slight shift in T_onset_ and a decrease in the maximum degradation rate are observed. These effects may be related to the multilayer architecture and the presence of cellulose acetate (CA) layers. In addition, weak interfacial interactions, such as hydrogen bonding between PLA carbonyl groups and residual hydroxyl groups of CA, cannot be completely ruled out. However, FTIR and DSC analyses did not provide direct evidence of significant intermolecular interactions, and therefore, this effect should be regarded as a possible contribution rather than a confirmed mechanism [48,49]. The char-forming contribution of CA is likely a more relevant factor, as the higher carbonaceous residue generated by CA increases the overall char yield of the multilayer structures, particularly in CA-rich configurations (e.g., CA/PLA/CA). This behavior is consistent with the known pyrolytic pathways of cellulose derivatives [50]. The residual mass at 700 °C follows the order: CA > CA/PLA/CA > PLA/CA/PLA ≈ CA/PLA > PLA, demonstrating the dominant role of CA in char formation. However, the absence of significant upward shifts in T_max_ indicates that the multilayer design does not dramatically enhance intrinsic thermal stability but rather moderates degradation kinetics.

From a practical standpoint, all materials remain thermally stable up to approximately 300 °C, which is well above the typical processing temperatures of PLA-based materials (170–200 °C). Therefore, the incorporation of CA in multilayer configurations does not compromise processability. Moreover, the increased char yield observed in CA-containing systems may provide improved thermal resistance in applications where short-term exposure to elevated temperatures occurs. Similar trends have been reported for PLA/cellulose-based systems in the literature, where cellulose derivatives contribute to enhanced residual stability without altering the fundamental degradation mechanism [51,52].

Overall, the results demonstrate that multilayer structuring of CA and PLA yields thermally compatible systems characterized by single-step degradation, moderated degradation kinetics, and improved high-temperature residue compared to neat PLA, while preserving processing stability.

### 3.5. Mechanical Properties of Multilayer Materials

**Figure 8:**
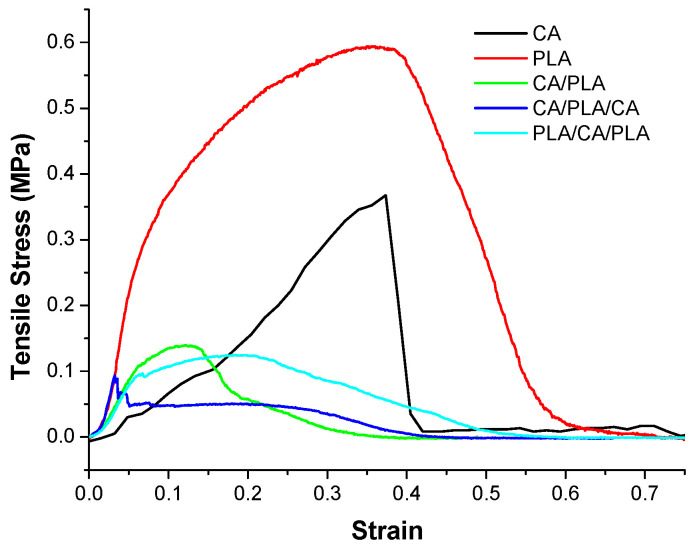
Representative tensile curves of the materials prepared by SBS.

In Figure 8, representative tensile curves of the materials prepared by SBS are shown. On the other hand, mechanical parameters extracted from the analysis of the tensile curves are gathered in Table 3. It can be seen that CA specimens (black curve) presented intermediate tensile strength (~0.3 MPa), the lowest rigidity (E ≈ 1.0 MPa), and relatively large strain before catastrophic drop (~0.4); PLA specimens (red curve) presented the highest tensile strength (~0.6 MPa) and rigidity (E ≈ 6.5 MPa), and large strain to failure (~0.5), being clearly stronger and tougher than CA materials. On the other hand, multilayer systems exhibit more complex mechanical behavior, which highlights the multifailure arising from the failure of each layer, as well as interface failure or delamination. In principle, each discontinuity observed in the tensile curves could be associated with a particular failure. The CA/PLA system (green curve) shows a low tensile strength (0.1 MPa), intermediate rigidity (E ≈ 2.3 MPa), and a small strain, even after considering the slow decay while breaking (~0.3), which allows considering this sample weak with relatively low ductility. The material CA/PLA/CA (blue curve) presents the lowest tensile strength (~0.08 MPa), in comparison with the rest of the materials, a relatively high rigidity (E ≈ 3.6 MPa), and a relatively large strain if the second failure mechanism, observed after the first stress drop, is considered (~0.4), which points out the poorest mechanical response in terms of toughness. Finally, the PLA/CA/PLA system (cyan curve) presents a moderate tensile strength (~0.1 MPa), an intermediate rigidity (E ≈ 2.1 MPa), and relatively high strain (~0.5). In addition, there is a correspondence between the size of the layers and the tensile strength observed; the higher the thickness of the layer, the higher the tensile strength, showing the lack of cooperative effect between the components in the multilayer materials. Only in the case of the system CA/PLA/CA is it observed that Young’s modulus approaches that with the highest value, suggesting that the PLA, the strongest material, efficiently transfers the load to the outer layers of CA, which causes them to reach their failure sooner at a strain of ~0.05.

As can be observed (Figure 8, Table 3), the mechanical behavior of the SBS-prepared materials can be reasonably explained by the fact that, beyond the nature and the composition of the materials, morphology and layer architecture (Figure 5) play a key role. Although all samples exhibit very high porosity (~85–92%), their tensile response varies significantly, indicating that mechanical performance is governed primarily by fiber organization, layer continuity, and interfacial integrity rather than porosity alone. Single-layer CA films with a morphology consisting of a dense polymer matrix permeated by numerous irregular, interconnected pores result in low stiffness (E ≈ 1.0 MPa), intermediate tensile strength (~0.3 MPa), and moderate strain at failure. In contrast, PLA mats exhibit a more homogeneous and entangled fiber network, which promotes efficient stress transfer and explains their markedly higher stiffness (E ≈ 6.5 MPa), tensile strength (~0.65 MPa), and strain to failure, confirming PLA as the strongest and toughest material in this study. Multilayer systems show more complex mechanical responses, characterized by multiple stress drops associated with layer failure and interfacial delamination. The substantially lower tensile strength of the multilayer systems compared with the individual PLA and CA layers indicates that the interfaces become the mechanically weakest regions of the structure. Under tensile loading, stress must be transferred across the CA/PLA interfaces for the different layers to deform cooperatively. However, because the SBS process generates distinct layers with limited fiber interpenetration and no evidence of strong interfacial bonding, stress transfer is inefficient. As a result, local debonding and interfacial crack initiation occur at stresses well below the intrinsic strength of the constituent layers. Once interfacial failure begins, the layers no longer share the applied load effectively, causing premature failure of the multilayer assembly and tensile strengths that are lower than those of either individual material. The CA/PLA bilayer presents low tensile strength and limited ductility, consistent with the sharp morphological contrast between layers and weak interfacial cohesion. The CA/PLA/CA configuration shows increased stiffness but the lowest tensile strength, as the outer CA layers fail prematurely and prevent effective load sharing with the PLA core, leading to poor toughness despite relatively large overall strain. Placing the stronger component at the outer layers (PLA/CA/PLA) improves mechanical performance, yielding moderate tensile strength, intermediate stiffness, and high strain at break. This architecture delays catastrophic failure by allowing the outer PLA layers to bear most of the applied load, even though interfaces remain mechanically weak. Overall, tensile strength increases with the thickness and placement of the mechanically dominant PLA layers, demonstrating the absence of synergistic reinforcement in the multilayer systems. The lack of fiber interpenetration and weak interfacial adhesion limit cooperative effects, causing each layer to behave largely independently under load. These results highlight the critical role of SBS-induced morphology and layer arrangement in controlling the mechanical response of multilayer fibrous materials. Therefore, mechanical performance is expected to be improved by increasing fiber interpenetration, for example, by looking for conditions to make the CA solution spinnable to obtain fibers and enhancing interfacial adhesion by surface functionalization or the addition of compatibilizers.

### 3.6. Water Vapor Permeability

Table 4 gathers results obtained from the water vapor permeation tests. All the values fall in a narrow range (889–922 g·m^−2^), meaning that all samples are highly permeable to water vapor, confirming their breathable nature. This result is consistent with the high porosity and open fibrous morphology observed in the SEM micrographs and with the characteristics of each component, CA and PLA, respectively, where CA layers present more open voids leading to higher permeation, while PLA layers, still permeable, have fibers that pack somewhat more densely, leading to slightly lower permeation. In addition, these values are significantly higher than those typically reported for conventional commercial food-packaging films such as polyethylene (PE), polypropylene (PP), or PET, which are designed as moisture barriers and exhibit WVP values one to two orders of magnitude lower [53].

In contrast to the highly porous SBS-derived CA/PLA systems presented here, recent biodegradable multilayer and composite films generally exhibit lower water vapor permeability but with stronger barrier performance, highlighting distinct application areas. While such low permeability is advantageous for dry or moisture-sensitive products, it is often detrimental for fresh and respiring foods, where moisture accumulation and condensation accelerate microbial growth and quality loss.

For example, PLA films with coatings or additives such as layered clay and chitosan show WVTR values around 184–222 g·m^−2^·day^−1^ for neat PLA and values that are even lower when active coatings are applied [54,55], indicating enhanced barrier performance compared with pure PLA but still orders of magnitude below the high permeability of SBS mats (890–920 g·m^−2^·day^−1^) reported in this study, which favors breathability rather than blockage of moisture transfer. Other materials based on nanocellulose composites or PBAT blends also aim to reduce WVTR; for instance, PBAT nanocomposites obtained by melt mixing have achieved WVTR values as low as ~389 g·m^−2^·day^−1^ [56], approximately half of some conventional PLA films, which improves barrier performance, but this is still significantly lower than the SBS porous films. Other biodegradable multilayer systems produced by electrospinning have been shown to improve mechanical cohesion and functional performance compared with simple monolayer films. For instance, multilayer films combining poly(lactic acid) with nanofiber interlayers of fish gelatin demonstrated substantially lower oxygen and water vapor permeability than the corresponding monolayer films, along with increased tensile strength, confirming that multilayer architectures can significantly enhance barrier and mechanical properties in biodegradable packaging materials [57]. Thus, compared with other multilayer biodegradable systems that focus on maximizing barrier properties, the SBS CA/PLA materials in this work represent a distinct functional class optimized for breathable packaging applications, where high WVTR is advantageous for fruits and vegetables rather than moisture barrier dominance.

Here, it is interesting to highlight that although oxygen permeability measurements were not performed in this study, water vapor permeability results provide relevant insight into the breathable nature of the materials. However, future studies should be done in order to address gas transport properties in greater detail.

## 4. Conclusions

Porous cellulose acetate (CA), poly(lactic acid) (PLA), and CA/PLA multilayer films were successfully fabricated by solution blow spinning (SBS), demonstrating the versatility of this scalable technique for producing biodegradable packaging materials with tailored architectures. Sequential deposition enabled well-defined single-layer and multilayer structures while preserving high porosity across all configurations. ATR-FTIR, SEM, DSC, and TGA analyses consistently showed that CA and PLA layers retain their chemical identity, morphology, and intrinsic thermal behavior within the multilayer systems. The absence of spectral changes, thermal transition shifts, or altered degradation profiles confirms negligible polymer interdiffusion or specific intermolecular interactions, indicating that interlayer integration occurs mainly through physical contact and void filling rather than molecular mixing. Morphological analysis revealed open, interconnected porous structures for CA and predominantly fibrous networks for PLA, with clear layer boundaries in multilayer architectures. These features directly influence the mechanical and transport properties of the materials. Mechanical testing showed that PLA provides higher stiffness and strength, whereas CA contributes greater compliance. Multilayer systems exhibited complex tensile responses governed by interfacial failure, highlighting that mechanical performance can be tuned through layer arrangement rather than synergistic reinforcement. All materials displayed high and narrowly distributed water vapor permeability, largely independent of layer sequence, confirming that breathability is governed by the porous SBS morphology. The permeability values exceeded those of conventional packaging films, making these materials particularly suitable for breathable food packaging applications. Overall, SBS-derived CA/PLA multilayer fibrous films combine high moisture permeability, adjustable mechanical behavior, and full biodegradability using food-contact-approved polymers.

Despite these promising results, the mechanical performance of the multilayer systems was limited by weak interfacial adhesion between CA and PLA layers, resulting in poor stress transfer and premature interfacial failure under tensile loading. Therefore, future efforts should be focused on improving interlayer bonding through strategies such as surface activation treatments (e.g., plasma or corona treatment), compatibilizer incorporation, or chemical surface modification of the fibers. Additionally, promoting fiber interpenetration during sequential SBS deposition may enhance load transfer across interfaces and improve the overall mechanical integrity of the multilayer structures. These approaches could further expand the applicability of SBS-derived biodegradable multilayer materials for packaging applications requiring a better balance between permeability and mechanical performance. However, this work demonstrates a viable route to sustainable, industrially scalable packaging materials that bridge the gap between dense barrier films and low-performance breathable systems, offering strong potential for packaging fresh and short-shelf-life food.

## Figures and Tables

**Table 1:** Sample codes, polymer components used, and schemes showing the configuration of the samples prepared and their thickness.

Sample	CA	PLA	Scheme	Thickness (µm)
CA	x		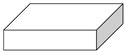	184 ± 2
PLA		x	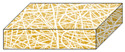	405 ± 3
CA/PLA	x	x	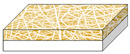	285 ± 14
CA/PLA/CA	2x	x	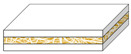	456 ± 32
PLA/CA/PLA	x	2x	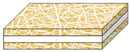	598 ± 11

**Table 2:** Differential scanning calorimetry data for the first heating scan.

Sample	T_g_ (°C)	T_m_ (°C)	χ (%)
CA	-	-	-
PLA	68.9	166.54	26.1
CA/PLA	69.5	166.73	19.7
CA/PLA/CA	69.4	168.46	17.8
PLA/CA/PLA	69.7	167.98	29.6

**Table 3:** Mechanical parameters extracted from the tensile test curves and values of porosity.

Sample	E (MPa)	σ_max_ (MPa)	ε	Porosity (%)
CA	1.03 ± 0.37	0.296 ± 0.07	0.35 ± 0.04	85.6 ± 0.6
PLA	6.48 ± 3.13	0.656 ± 0.18	0.55 ± 0.09	90.6 ± 0.3
CA/PLA	2.29 ± 0.76	0.113 ± 0.06	0.34 ± 0.03	90.2 ± 0.6
CA/PLA/CA	3.62 ± 1.26	0.084 ± 0.02	0.45 ± 0.09	91.6 ± 0.7
PLA/CA/PLA	2.12 ± 0.446	0.122 ± 0.01	0.52 ± 0.03	90.5 ± 0.1

**Table 4:** Results obtained from the water vapor permeation tests.

Sample	WVTR (g/m^2^·Day)
CA	921.91 ± 4.50
PLA	895.83 ± 3.49
CA/PLA	914.35 ± 6.57
CA/PLA/CA	906.31 ± 8.98
PLA/CA/PLA	889.25 ± 4.47

## Data Availability

The original contributions presented in this study are included in the article. Further inquiries can be directed to the corresponding author.
